# Fractal modes and multi-beam generation from hybrid microlaser resonators

**DOI:** 10.1038/s41467-018-04945-8

**Published:** 2018-07-03

**Authors:** José A. Rivera, Thomas C. Galvin, Austin W. Steinforth, J. Gary Eden

**Affiliations:** 1 Laboratory for Optical Physics and Engineering, Department of Electrical and Computer Engineering, University of Illinois, Urbana, IL 61801 USA; 2 Department of Bioengineering, University of Illinois, Urbana, IL 61801 USA; 3 Present Address: Lawrence Livermore National Laboratory, Livermore, CA 94550 USA

## Abstract

Fractals are ubiquitous in nature, and prominent examples include snowflakes and neurons. Although it has long been known that intricate optical fractal patterns can be realized with components such as gratings and reflecting spheres, generating fractal transverse modes from a laser has proven to be elusive. By introducing a 2D network of microspheres into a Fabry-Pérot cavity bounding a gain medium, we demonstrate a hybrid optical resonator in which the spheres enable the simultaneous generation of arrays of conventional (Gaussian) and fractal laser modes. Within the interstices of the microsphere crystal, several distinct fractal modes are observed, two of which resemble the Sierpinski Triangle. Coupling between adjacent fractal modes is evident, and fractal modes may be synthesized through design of the microsphere network. Owing to a unique synergy between the gain medium and the resonator, this optical platform is able to emit hundreds of microlaser beams and probe live motile cells.

## Abstract (web summary)

Although fractal optical patterns have been realized previously, the direct generation of fractal modes from a laser has proven challenging. Here, Rivera et al. modify an optical cavity with a microsphere array to produce fractal laser modes and probe live cells, which serve as a component of the resonator.

## Introduction

In the six decades since Schawlow and Townes^1^ predicted the realization of lasers (optical masers) comprising a gain medium combined with a plane-parallel (Fabry-Pérot) cavity, optical resonators have taken on a stunning variety of forms. Although optical cavities generally serve to extend the effective length of the gain medium (and, thus, the time available for the buildup of the optical field intensity), they also define the transverse modes of the resonator. Owing to the relationship between the transverse refractive index profile of the gain medium in an operating laser and the quality (*M*^2^ value) of the resulting beam, conventional resonators would benefit from the ability to generate hundreds of parallel microlaser beams from an aperture of modest dimensions. Pixelating the plane transverse to the axis of the optical cavity would afford the opportunity to synthesize beam wavefronts in the far-field having an arbitrary phase profile, and constrain diffractive losses. Beyond multiple-beam generation, however, lies the potential for manipulating transverse modes in a reconfigurable intra-cavity environment, with emphasis on realizing fractal laser modes and controlling their formation^2–12^.

We report here the transformation of the properties of a laser medium bounded by a plane-parallel optical resonator, through the introduction of a two-dimensional array of microspheres onto (or in proximity to) the surface of one cavity mirror. With this geometry, both fractal laser modes and the Hermite-Gaussian (HG), Laguerre-Gaussian (LG), and Ince-Gaussian (IG) transverse modes characteristic of stable resonators are observed from a single cavity. Given the proper sphere diameter and mirror spacing, the optical resonator is stabilized along axes parallel to the cavity axis and defined by the lateral position of each sphere. With a colloidal quantum dot (QD) solution serving as the gain medium and 20–200 μm diameter spheres, stable cavities result for mirror spacings *L* up to approximately twice the sphere diameter. For cavity lengths below the stability limit, a plethora of complex transverse modes is produced, and previously unreported IG_*mn*_ modes with indices *m,n* > 30 are generated routinely.

Fundamental and higher-order fractal laser modes are produced in regions encompassed, or partially bounded, by three or more microspheres. By modifying the trigonal pyramid optical scattering geometry of Sweet, Ott, and Yorke^13^ and providing a gain medium and two auxiliary mirrors, we observe fractal intensity patterns not reported previously—fractal transverse eigenmodes generated directly by a laser^8–12^. Associated with three and four microsphere topologies, the fractal mode emission emanates from the interstices in the sphere network and is the result of a unique synergy between the resonator and gain medium. Fresnel diffraction of the pump optical field by the microspheres bordering an interstice results in interference fringes produced in the gain medium in which the microsphere array is immersed. Intersections between these spherical surfaces of peak optical gain serve as a scaffold from which the fractal modes are constructed. Precise placement of the microspheres on the surface of one cavity mirror allows for particular fractal modes to be specified, and the sequential construction of higher-order modes from the fundamental to be viewed as the pump optical power is increased. Four distinct fractal laser modes are observed repeatedly, two of which closely resemble the Sierpinski Triangle or Fresnel diffraction simulations. Self-similarity extending over three spatial scales is observed in experimental images, recorded with a 20 × microscope objective, which confirms the fractal nature of the transverse modes. Although the primary mechanism responsible for generating the fractal modes appears to be linear in the pump optical field intensity, the influence of nonlinearities, such as the optical Kerr effect, cannot be discounted. Coupling of fractal laser modes in a plane transverse to the optical axis is enabled by slight separations between adjacent spheres, and has also been observed for the first time.

With a hexagonal array of close-packed, 80 μm diameter spheres, an array of 172 microlasers has been demonstrated in which each Gaussian microbeam has a mode volume as small as 2 pL. Each beam has a fundamental mode transverse intensity profile and the aperture for the microlaser array is < 1.5 mm. The microrefractive element(s) serving to stabilize the optical resonator need not be either a microsphere or inanimate, and lasing has been observed by stabilizing a Fabry-Pérot resonator with *Chlamydomonas reinhardtii*, a unicellular organism often adopted as a model for studying ciliary disease^14^ and light-sensitive proteins^15^. When live, motile *C. reinhardtii* cells enter the pump optical field in a double-chambered resonator, the organism is temporarily a refractive element in the optical system, and the transverse mode intensity maps of the resulting laser emission are determined by the orientation and structure of the cell. Such an approach to the coherent imaging of biological organisms in vivo is potentially capable of resolving both three-dimensional structure and phototactic behavior on the nanosecond time scale.

## Results

### Generation of Gaussian and fractal transverse modes

**Fig. 1:**
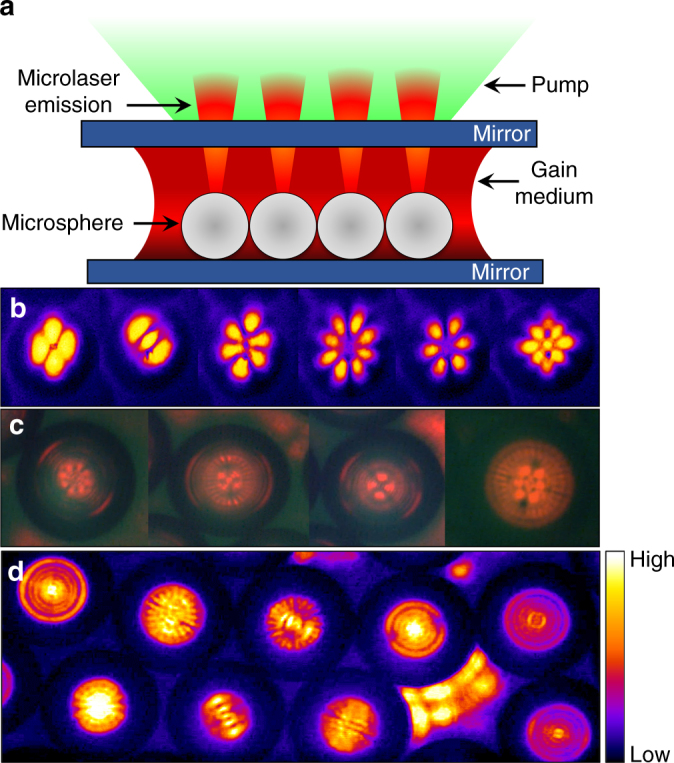
Hybrid microlaser resonators. **a** Cross-sectional diagram (not to scale) of one configuration of the hybrid optical resonator. **b**, **c** Images of frequently observed IG_*mn*_ modes associated with a sphere-stabilized microlaser. **d** Optical micrograph of a portion of an array of microspheres, illustrating (in false color) various IG transverse modes as well as lasing within several interstitial regions. For the images of **b**–**d**, all of the microspheres have a diameter of 80 μm

One configuration of a hybrid optical resonator is illustrated in cross-section (not to scale) in Fig. 1a. A plane-parallel cavity, comprising two highly reflecting (*R* > 99.9% at 650 nm) mirrors, confines a liquid or solid-state gain medium, and kinematic optical mounts allow for the separation (*L*) between the mirrors to be varied in increments as small as 0.5 μm. For all of the experiments described here, the gain medium is an 8 μM solution of colloidal ZnS/CdSe QDs having carboxyl functional groups which enable the dots to be soluble in water. Note that the 2D network of polystyrene microspheres is immersed in the liquid gain medium—further details can be found in the Methods section.

When the gain medium and microspheres are flood-illuminated through the upper mirror (Fig. 1a) with frequency-doubled, Nd:YAG laser (*λ* = 532 nm) pulses, aqueous solutions of ZnS/CdSe QDs having peak gain near 660 nm yield a broad range of Gaussian transverse modes associated with the microlaser beams. Several of the lower-order transverse modes observed most frequently are displayed in false color in Fig. 1b. All were recorded for 80 μm diameter (*d*) spheres and are IG_*mn*_ eigenmodes, which are solutions of the paraxial wave equation in elliptic coordinates^16^. Examples include the ${\mathrm{IG}}_{3,3}^{\mathrm{e}}$ and ${\mathrm{IG}}_{4,4}^{\mathrm{o}}$ modes of Fig. 1b. Combining the large gain coefficients ( > 100 cm^−1^) available from optically-pumped QD solutions with the Q of the present resonator enables IG and LG modes not reported previously to be produced readily. Figures 1c and d show a few of the transverse modes observed with a 10 × objective in the laser microscope, *L* *=* 140 μm, and *d* = 80 μm microspheres. Multiple IG modes have been reported previously^17^ in resonators pumped off-axis or intentionally misaligned but the mode order did not exceed 20. Numerous modes for which the indices *m* or *n* are > 30 have been observed and, for example, the hybrid (“phi”) mode presented in Supplementary Fig. 1 is a superposition of the ${\mathrm{IG}}_{33,33}^{\mathrm{e}}$and ${\mathrm{IG}}_{21,1}^{\mathrm{o}}$modes. The panel at right in Supplementary Fig. 1 is a simulation of a linear combination of these modes and, for clarity, both are illustrated as lying in the plane of the page. An assortment of other transverse modes observed in these experiments is also presented in the Supplementary Figs. 2–4. The data of Fig. 1 and Supplementary Figs. 1–4 demonstrate directional emission from a QD laser, and the Gaussian resonators of Fig. 1b–d are the smallest yet achieved for colloidal QD optical oscillators (mode volumes ≤ 2 pL). In addition to the LG or IG modes correlated with individual microspheres, lasing is also occurring in Fig. 1and Supplementary Figs. 2, 3 and 5 at interstitial sites in the microsphere network. As discussed below, the laser mode patterns produced within such gaps between spheres are fractal in nature.

### Fractal modes for three and four microsphere topologies

We observe fractal laser modes in the interstices of a microsphere array that are encompassed by three or more spheres. Owing to the reflectivity of polystyrene or silica spheres at grazing incidence, diffraction losses in the resonator of Fig. 1a are reduced in the vicinity of the sphere/water (gain medium) interface and fractal modes appear. Furthermore, the fractal modes associated with specific microsphere arrangements appear to be unique to that geometry. Both three and four sphere topologies have been examined and, for interstitial regions encompassed by three spheres, four distinct fractal laser modes have been observed. Two of these resemble the Sierpinski Triangle and one matches the prediction of Fresnel diffraction simulations. The ability afforded by microfabrication techniques to position microspheres precisely on a mirror surface allows for a desired fractal mode to be produced and to pattern the requisite sphere arrangement over substantial areas.

**Fig. 2:**
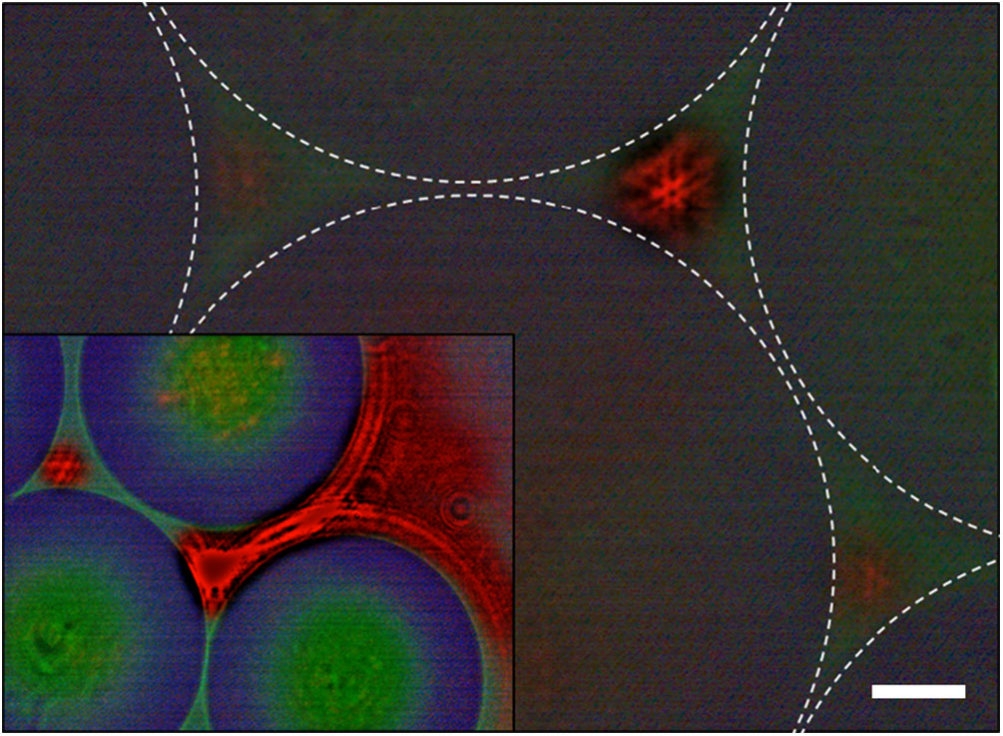
Optical image of a microlaser fractal transverse mode produced at the intersection of three, 200 μm diameter polystyrene spheres. The spheres are immersed in a QD solution, *L* = 370 μm, and the micrograph is a single pump pulse image recorded with a 20 × objective. The white bar at lower right indicates a length of 25 μm. The inset is a representative image of laser mode coupling facilitated by increasing the separation between two spheres

A light scattering system devised by Sweet et al.^13^, comprising reflecting spheres arranged in the form of a trigonal pyramid, yielded fractal patterns in the light intensity distribution emerging from any of the four optical ports (“basins”). In the present experiments, the top sphere of the ref.^ 13^ pyramid has been removed, and a symmetric resonator is realized in which microsphere ensembles in the form of quasi-triangles, rectangles, etc. are positioned between two mirrors (Fig. 1a). Figure 2 is a representative microscope image of a portion of an array of 200 μm diameter spheres within the resonator of Fig. 1a. Recorded in plan view (oriented toward the bottom mirror) with a 20 × microscope objective, this micrograph was acquired with a single pulse of the pump laser, and the spatial scale at lower right indicates a length of 25 μm. The interstice at upper right (where dashed white circles denote the perimeters of the three bordering spheres) shows a laser fractal mode, and a second, weaker mode is developing in the inter-sphere gap at lower right. The dominant feature of the stronger transverse laser mode comprises two superimposed triangles forming a “Star of David” pattern, lying at the junction of three larger triangles separated azimuthally by 120^o^. Another unanticipated aspect of the experimental results reported here is that of coupled fractals, illustrated by the inset to Fig. 2 (lower left). In this case, two of the spheres have been separated intentionally and the extension of laser radiation beyond the 3-sphere interstice is evident.

**Fig. 3:**
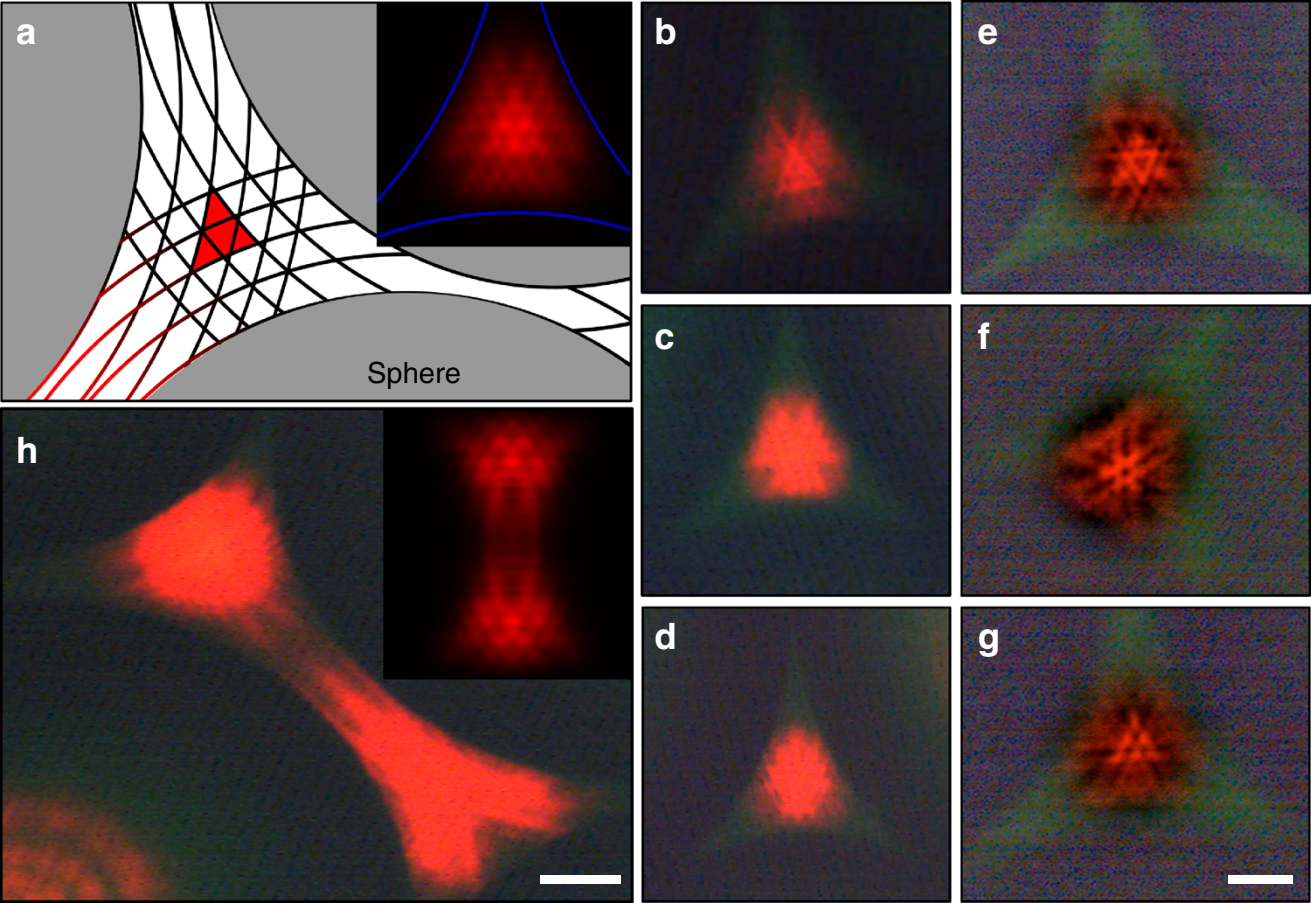
Observed and calculated fractal laser modes for three and four microsphere topologies. **a** Illustration of a 3-sphere arrangement and its associated triangular fundamental laser mode (in red). The inset is the calculated modal intensity map, shown in false color. **b**–**d** Progression of laser mode development from an early stage to more fully developed fractals (the interiors of **c** and **d** are saturated), recorded with a 10 × objective. **e**–**g** Fractal laser modes observed with a 20 × microscope objective. **h** Optical micrograph of an open interstitial region, illustrating fractal laser mode coupling. The inset is a simulation of the interaction between fractal modes produced by a 4-sphere arrangement

Panel a of Fig. 3 illustrates qualitatively the interstitial region lying between three adjacent spheres, and the interference fringes produced by Fresnel diffraction of the pump optical field by each of three microspheres. Representing spherical surfaces of peak optical gain in the QD/solvent gain medium, the network of intersecting fringes provides a scaffold for the fractal mode as it is constructed. Consequently, the geometry of laser fractal modes is dependent upon the precise microsphere arrangement.

Four distinct fractal laser modes have been observed experimentally for the 3-microsphere topology. In initial experiments, images were acquired with a 10 × objective in the laser microscope and several examples are given in panels b–d of Fig. 3. Each image was recorded with a single pump laser pulse, and it is immediately clear that the observed modes are not Gaussian. Rather, the transverse mode of Fig. 3b closely resembles the calculated intensity map shown by the inset to Fig. 3a. As described in detail in Methods, numerical simulations of the eigenmodes expected for Fresnel diffraction were carried out by calculating the first Rayleigh-Sommerfeld integral. Notice that the micrograph of Fig. 3b confirms the existence of the four largest triangles in the calculated image but also suggests the presence of the three brightest features at the core of the simulated mode. Figures 3c and d illustrate the subsequent construction (at higher pump intensities) of the fractal network by the addition of scaled replicas of the fundamental^10, 11^. The self-similarity of fractal laser modes results from the superposition of de-magnified versions of the fundamental^2–10^.

Supplementary Fig. 5 shows fractal laser emission from several interstitial regions in which fractal mode development was arrested at the stage of Fig. 3c. However, successive increases in the complexity of the fractal network generate a variety of mode patterns, such as that given in Fig. 3d. It should be mentioned that the mode perimeter in Fig. 3d, for example, comprises a series of de-magnified triangles, thus giving the intensity pattern a serrated appearance^13^. The “sawtooth” perimeter of the modes of Fig. 3c and d is a preliminary indication that self-similarity spans more than an order of magnitude on the spatial scale.

In an effort to improve spatial resolution, more recent fractal mode images were recorded with a 20 × objective and processed by ImageJ software which introduces a Gaussian band-pass filter in the Fourier (spatial frequency) domain, thereby improving contrast (Supplementary Fig. 8). With this enhanced imaging capability, four basic mode intensity patterns are observed repeatedly, and a sampling of three of these is given in panels e–g of Fig. 3. Of particular interest is the mode of Fig. 3g whose central triangle bears a remarkable resemblance to the Sierpinski Triangle (or Sierpinski Sieve) which is an exact fractal. Although the largest interior triangle of the Sierpinski geometry is absent in 3g, smaller triangles defining the boundary of this central triangle are now clearly resolved. Indeed, Fig. 3g appears to be the same mode predicted by the calculations (inset, Fig. 3a) and is actually more fully resolved than either the simulation or Fig. 3b.

The other most commonly observed modes, shown in panels e and f of Fig. 3 and Supplementary Fig. 6, represent other fractal variants. Figure 3e, for example, has a central Sierpinski-like triangle comprising a bright inverted triangle and three features (presumably unresolved triangles) forming the corners of the larger triangle. Panel f of Fig. 3 is the same mode as that presented in Fig. 2. As noted previously, triangles of several spatial scales are observed, and similar comments can be made for the final mode given in Supplementary Fig. 6. It must be emphasized that images b–g of Fig. 3 are formed at the intersection of three, 200 μm diameter spheres, and the mean gap distance is on the order of 25 μm. Therefore, the smallest resolved features in these images, as well as Fig. 2 and Supplementary Figs. 6 and 7, are ~1 μm in extent (i.e., near the diffraction limit). A set of optical micrographs corresponding to those of Fig. 3 but recorded for a 4-sphere interstitial topology are given in Supplementary Fig. 7. For this geometry, the fundamental mode is square and, with increasing pump intensity, the mode profile expands as de-magnified copies of the fundamental are superimposed. Finally, calculations of the fractal dimension *D* with FracLac/ImageJ software show that 1.4 ≤ *D* ≤ 1.7 for the images of Figs. 2 and 3.

Insofar as the origin of these modes is concerned, the ability of Fresnel diffraction simulations to predict the general features of Fig. 3b and g suggests that the primary mechanism responsible for the fractal patterns is linear in the optical field intensity. However, it is not possible at present to dismiss the influence of a nonlinear process, particularly in view of the synergy between the optical resonator and gain medium to form the pump interference fringes which define the geometry of the fundamental modes and all subsequent fractals. The centrality of Fresnel diffraction of the pump field to fractal laser mode formation was confirmed by additional experiments in which the sphere network was replaced by an array of hemispheres. Although these experiments were identical in every other respect to those of Figs. 1–3, fractal modes were not observed.

A second point to be made is that the fractal laser mode in any given interstice is surprisingly stable and reproducible from “shot-to-shot” of the pump laser, in contrast to the relative instability of the high-order LG modes of Fig. 1. Although gain saturation is one nonlinear optical mechanism that potentially influences the appearance of fractal modes, calculations indicate that its impact is small. Photoluminescence measurements of the upper laser level lifetime of the gain medium with ~5 ns excitation pulses yielded 44 ± 3 ns, from which the stimulated emission cross-section (*σ*_se_) is found to be 2 × 10^−16^ cm^2^. Consequently, *I*_sat_ for this homogeneously broadened gain medium is approximately 36 kW/cm^2^, which is at least a factor of 3 higher than the estimated peak intensity associated with Fig. 3 and Supplementary Figs. 5–7. Similar comments appear to be valid for the optical Kerr effect, and more extensive data will be required to determine the potential influence of nonlinear processes on the generation of fractal laser modes.

Before leaving this subject, it should be mentioned that the interaction of fractal laser modes has also been observed in multi-sphere ensembles in which a gap exists between at least two of the spheres (i.e., an “open” topology). As an example, Fig. 3h is a micrograph of an interstitial region bounded by four spheres of the same diameter (*d* = 200 μm). Coupling between the fractal modes generated within neighboring 3-sphere interstices is occurring and the intensity pattern resembles “viscous fingering” fractals observed in fluids^14^. Application of the Fresnel diffraction simulations described earlier to the geometry of Fig. 3h yields the intensity map shown by the inset to panel h of the figure. Again, the general features of the experimental image are reproduced. Such fractal mode interactions are observed frequently (compare, for example, the insets of Figs. 2 and 3a) and are accessible only because the two-dimensional microsphere array can be patterned to have virtually any desired inter-sphere topology.

### Individual microsphere-stabilized resonator characteristics

**Fig. 4:**
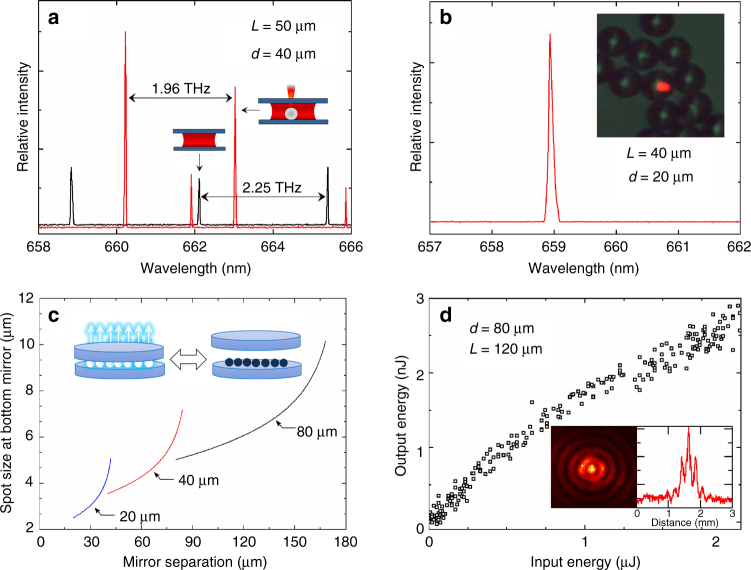
Summary of the properties of microsphere-stabilized microlaser arrays. **a** Comparison of the laser spectra recorded for the resonator of Fig. 1a in which a single 40 μm sphere rests on the lower mirror, with the same resonator but in the absence of a microsphere. **b** Single-mode spectrum observed for a *d* = 20 μm microsphere when *L* = 40 μm. The inset is a microphotograph of one lasing sphere in the midst of other spheres. **c** Stability diagram for *d* = 20,40,80 μm diameter spheres showing the variation with *L* of the laser spot size at the lower mirror. **d** Dependence of the output pulse energy on the 532 nm optical pump pulse energy for a single 80 μm diameter sphere. The inset is the laser intensity map for the single optical oscillator in the far-field, along with its intensity lineout

In Fig. 4a, a laser spectrum representative of those observed when a single microsphere (*d* = 40 μm) is situated on the lower mirror, is compared with the corresponding spectrum recorded when no sphere is present. In the absence of a microsphere (black trace, Fig. 4a), adjacent longitudinal modes are separated by 2.25 THz, which is precisely the free spectral range (FSR) expected of a Fabry-Pérot cavity for which *L* = 50 μm and *n* = 1.33 [water, 293 K]. When a microsphere is introduced to the resonator, the FSR decreases to 1.96 THz because a polystyrene sphere (*n*_s_ = 1.59) has displaced a small volume of water in the cavity. An accurate calculation of the revised FSR requires that one account for the resonances arising from Fresnel reflection at normal incidence to the microsphere–water interface.

Numerical simulations treating the microsphere within the Fabry-Pérot resonator as two coupled cavities accurately predicts the decrease in FSR depicted in Fig. 4a. By raising the resonator FSR with short cavities (*L* < 100 μm), single longitudinal and transverse mode operation of a microsphere oscillator was achieved, and Fig. 4b is an example of a spectrum acquired for *L* *=* 40 μm. The inset to Fig. 4b is an optical micrograph of several 20 μm spheres lying on the surface of a highly reflecting mirror and immersed in a QD solution. The pump was passed through an aperture so that only one microsphere would lase, and it should be noted that the measured spectral width of the mode of Fig. 4b is instrument-limited.

The ordered pairs of *L* and *d* for which the resonators are stable were determined by ABCD matrix theory, and the results of calculations for polystyrene microspheres with diameters of 20, 40, and 80 μm are presented in Fig. 4c. The diameter of the beam waist at the lower mirror is given only for those regions of mirror separation and microsphere diameter at which the optical cavities are stable. For polystyrene spheres in water, the cavity becomes unstable for values of *L* slightly exceeding twice the sphere diameter. Experiments have verified the behavior depicted in Fig. 4c. Specifically, the termination of lasing at the extrema occurs over a narrow range in mirror separation. Measurements show the transition from vigorous lasing in the resonator to an inability to obtain lasing (regardless of pump power) occurs in a Δ*L* < 3 μm interval.

In Fig. 4d, the dependence of the laser output pulse energy on the pump pulse energy is shown for a single microsphere. The threshold pump energy is ~50 nJ, and the far-field image of the microsphere laser emission is the left portion of the Fig. 4d inset. The intensity lineout associated with this diffraction image, also presented in Fig. 4d, yields a fringe pattern with a modulation depth > 70%.

### Multiple-beam generation and single-cell imaging

**Fig. 5:**
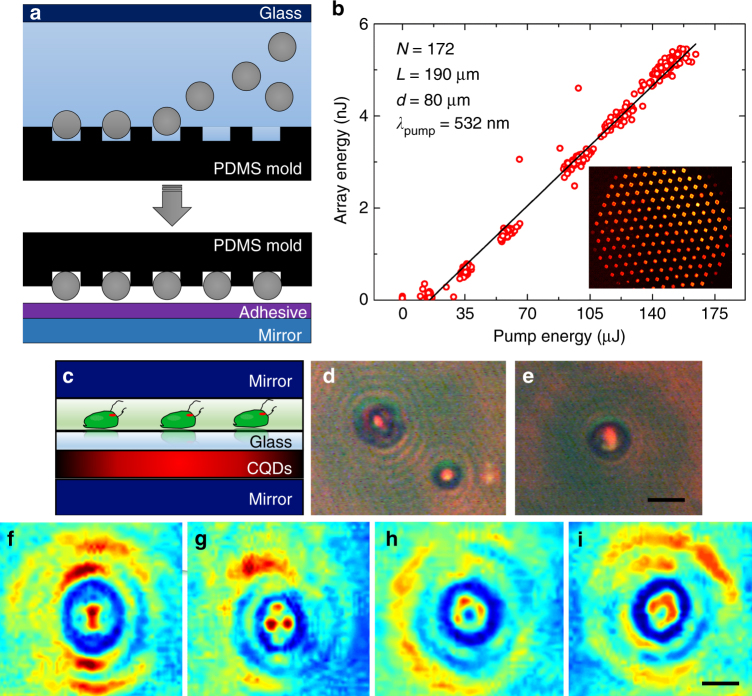
Resonators designed for multiple-beam generation with close-packed microsphere arrays or for the analysis of single cells in vivo. **a** Diagrams illustrating two steps in the placement of microsphere arrays onto the surface of a mirror with a PDMS template. **b** Dependence of laser output pulse energy on the pump (532 nm) pulse energy for an array of 172 microspheres. The inset is an optical micrograph of a portion of the hexagonal array while lasing, and the least-squares fit to the data is represented by the black line. **c** Cross-sectional diagram of a double-chambered resonator incorporating living *C. reinhardtii* cells. **d**, **e** Photographs of *C. reinhardtii* cells showing lasing in the cytoplasm of the unicellular organism. **f**-**i** False color versions of optical micrographs of *C. reinhardtii*, illustrating several observed laser mode patterns. Note the spatial scale (10 μm) in **i** and the interference fringes outside the cell

With a convective assembly and polydimethylsiloxane (PDMS) replica-molding process^18^, arrays of microspheres having precisely defined geometries were prepared on a template and subsequently affixed to the surface of the lower mirror with an optical adhesive. Two key steps in this process (see Methods), filling a PDMS mold with microspheres and transferring the sphere array to the mirror surface, are illustrated in Fig. 5a. Panel b of Fig. 5 summarizes measurements of the variation in laser output energy with pump energy (*λ* = 532 nm) for an array of 172 microlasers, generated with *d* = 80 μm spheres and having an overall beam “bundle” diameter of < 1.5 mm. The pump energy threshold is < 20 μJ/pulse which corresponds to < 120 nJ/sphere, and the black line represents the least-squares fit to the data. An optical micrograph of virtually all of the hexagonal array of spheres, recorded while lasing was in progress, is shown by the inset to Fig. 5b. The data of Fig. 5b demonstrate that 172 (and larger) microlaser arrays act as a single entity, and far-field images show that the beams add incoherently, which is advantageous for several applications. Although the optical-to-optical conversion efficiency and single pulse energies for this laser are both modest at present, changing the gain medium and inserting the oscillator of Fig. 1a into a master-oscillator-power amplifier (MOPA) configuration will undoubtedly improve the system performance significantly.

Although microspheres and other microrefractive elements are effective in stabilizing and pixelating a critically stable optical cavity, it must be emphasized that the microrefractive component need not be inanimate. That is, specific biological organisms and materials are perfectly capable of serving the same purpose. To illustrate this point, a double-chambered assembly was designed and positioned between two mirrors (Fig. 5c). The lower compartment was occupied by a gain medium (a QD solution), whereas living cells reside in the upper chamber. Initial studies have focused on *Chlamydomonas reinhardtii*, a motile single-cell microalgae exhibiting phototaxis and having a morphology approximating the geometry of a thin lens. Miccio et. al.^19^ have shown recently that red blood cells are also capable of acting as a microlens.

Figure 5d and e are optical micrographs showing *C. reinhardtii* cells at the moment a 532 nm pump pulse arrives. The entire area represented by the micrograph has been illuminated by the pump, and the cells are quasi-circular or elliptical (in plan view) with nominal diameters (or major axis lengths) of 10–20 μm. It is clear that lasing is occurring, and the transverse intensity profile for the microlasers associated with each of the cells in Fig. 5d and e is confined to the cytoplasm of the cell. As shown by Supplementary Movie 1, *C. reinhardtii* cells are drawn to the green pump radiation by their eyespot, and lasing occurs as long as a cell lies within the pump field. The false color images of Fig. 5f–i illustrate several of the two-dimensional, transverse intensity profiles that have been observed. Examination of hundreds of optical micrographs similar to those of Fig. 5d–i indicates that the mode profile varies in time, and reflects both the cell orientation and structure when a pump laser pulse arrives. This conclusion suggests that temporally resolved imaging of the central portion of these organisms is feasible by digital reconstruction, from the laser intensity profile, of the cells’ refractive index. Finally, circular interference fringes appearing outside the cells indicate the feasibility of also retrieving three-dimensional cell structure by in-line holography^20^.

## Discussion

Laser fractal modes for three and four microsphere waveguide topologies in a hybrid optical resonator have been observed for the first time, and the development of the fractal network with increasing optical gain (i.e., pump field intensity) has been captured. Four transverse laser fractal modes have been identified, and coupling of fractal modes has also been observed. Scores of microlaser beams have been generated from a Fabry-Pérot resonator of modest dimensions by pixelating the plane transverse to the longitudinal axis of the cavity with an array of microspheres. Stabilization of a plane-parallel laser cavity with a unicellular organism (*C. reinhardtii*) has also been demonstrated. Optical resonators stabilized by microspheres or other microrefractive elements provide the opportunity to control the phase and intensity profiles of a composite wavefront in the far-field, but the random phase variations of all the microlaser beams in the basic configuration (Fig. 1a) also suggest several intriguing applications. Early experiments, for example, indicate that multi-microbeam lasers are largely speckle-free when no effort is invested in locking the phases of individual beams.

## Methods

### Device fabrication, characterization, and imaging

Aqueous suspensions of microspheres (Duke 4000 Series Mono-sized Polymer Particles) were centrifuged for 1 min at 2000 rpm. After collecting 1 μl of the resulting pellet, spheres were mixed in a 1:10 ratio with an 8 μM solution of colloidal carboxyl-functionalized ZnS/CdSe QDs (Thermo Fischer Scientific, Inc.). Subsequently, 10 μl of the microsphere/QD mixture was pipetted onto the bottom mirror of a Fabry-Pérot resonator (*R* > 99% at 650–1100 nm for both mirrors; transmission of 90% at 532 nm). After dispensing a droplet of colloidal QDs, the cavity length was decreased using a precision linear translator until the droplet made contact with both mirrors. Parallelization of the mirrors comprising the resonator was ensured through interferometry with a He-Ne laser operating at 543 nm. Self-assembled arrays of microspheres, positioned at the bottom mirror, were imaged by a custom-built epifluorescence microscope while being flood-illuminated with ~8 ns pulses from a *Q*-switched, frequency-doubled Nd:YAG laser (PRF of 10 Hz). Spectra of microsphere-stabilized lasers were acquired by imaging the output of the microscope onto the entrance slit of a 0.75 m Czerny-Turner monochromator coupled to an intensified, charge-coupled device (CCD) camera (Princeton Instruments, Inc.). Experiments were conducted with both 10 × and 20 × objectives in the laser microscope in an effort to examine detail in the fractal mode profiles. The pump input and laser output energies for individual microspheres, as well as arrays, were recorded with calibrated pyroelectric or silicon detectors, respectively (Newport, Inc.).

### Convective assembly of microsphere arrays

PDMS templates comprising an array of cylindrical cavities (*d* = 80 μm) were prepared by a replica-molding process. After producing PDMS molds from silicon masters, they were oxidized by oxygen plasma for 1 min. The substrate was then placed under a glass slide that was aligned parallel to the surface. A solution of microspheres (Duke Scientific, Inc.) was dispensed between the glass slide and PDMS substrate. Dragging the meniscus of the solution over the hydrophilic surface at a constant velocity (100 μm/s) resulted in the convective assembly of spheres into close-packed hexagonal arrays. The substrate was allowed to dry at room temperature after deposition. After filling the holes of the PDMS array template with microspheres, a highly reflective mirror was coated with an optically clear adhesive tape having a thickness of 50 μm (OCA8146-2, ThorLabs, Inc.). The microsphere-filled PDMS substrate was placed above the coated mirror, with the spheres exposed to the surface, before gently pressing the substrates together. After peeling away the PDMS layer, arrays of microspheres remained affixed to the highly reflective mirror.

### Dual-chambered laser cavity for cell-stabilized lasers

A plane-parallel resonator having two internal compartments was constructed by first placing a spacer, having a thickness of 370 μm, onto a highly reflective mirror. A circular glass coverslip (thickness equal to 120 μm) was placed on top of the spacer after depositing a droplet of QDs onto the center of the mirror. A 10 μl droplet of *C. reinhardtii* cells (Carolina Biological, Inc.) was subsequently deposited onto the glass coverslip and another spacer was then placed on its surface. Finally, lowering another high reflector onto the top spacer completed the cavity. The two overlapping droplets were then pumped by the pulsed nanosecond Nd:YAG laser.

### Fractal mode calculations

Cavity eigenmodes of the interstitial lasing regions were calculated numerically with a modified Fox-Li iterative algorithm^21^. Inspired by Shen and Wang^22^, a two-dimensional fast-Fourier transform (FFT) method, incorporating the first Rayleigh-Sommerfeld diffraction integral, was developed for all field propagation calculations. The geometry of the simulated resonator was simplified, comprising a Fabry-Pérot cavity containing a uniform dielectric and a binary transmission mask lying in a plane parallel to the mirror surfaces and passing through the equatorial plane of all microspheres. The planar mask consists of opaque disks, one for each sphere and representing two-dimensional projections of the spheres onto the plane. Elsewhere, the mask is fully transmissive. After seeding the resonator with an initial optical field distribution, the simulations propagated the field through the cavity in an iterative fashion until a stable transverse mode was obtained. All calculated images presented here depict the mode profile at the surface of the mirror with which the microspheres are in contact.

### Data availability

All relevant data are available from the authors.

## Electronic supplementary material

Supplementary Information

Peer Review Report

Description of Additional Supplementary Files

Supplementary Movie 1
