# Supplementary material for: Fractal modes and multi-beam generation from hybrid microlaser resonators
Source: Nat Commun. 2018 Jul 3;9:2594. doi: 10.1038/s41467-018-04945-8 (PMC6030083; doi:10.1038/s41467-018-04945-8)
Supplement: Supplementary file 1 — Supplementary Information [file 41467_2018_4945_MOESM1_ESM.pdf]

Supplementary Information

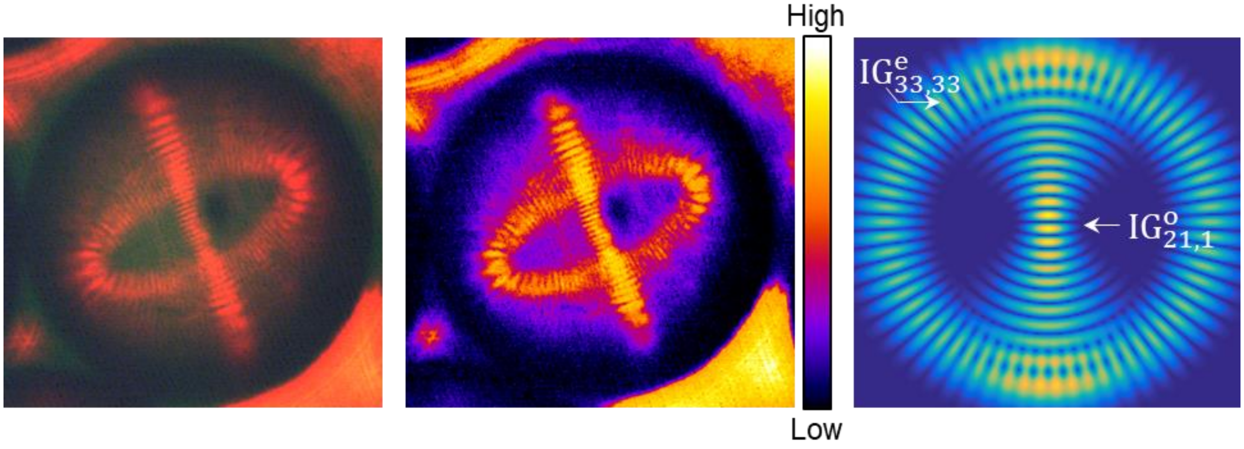

Supplementary Figure 1: Superposition of the $IG^{e}_{33,33}$ and $IG^{o}_{21,1}$ modes. These modes were observed in experiments with *d*=200 μm spheres (left, and false color at center). The panel at right is a simulation of the two modes, illustrated as a projection onto a plane.

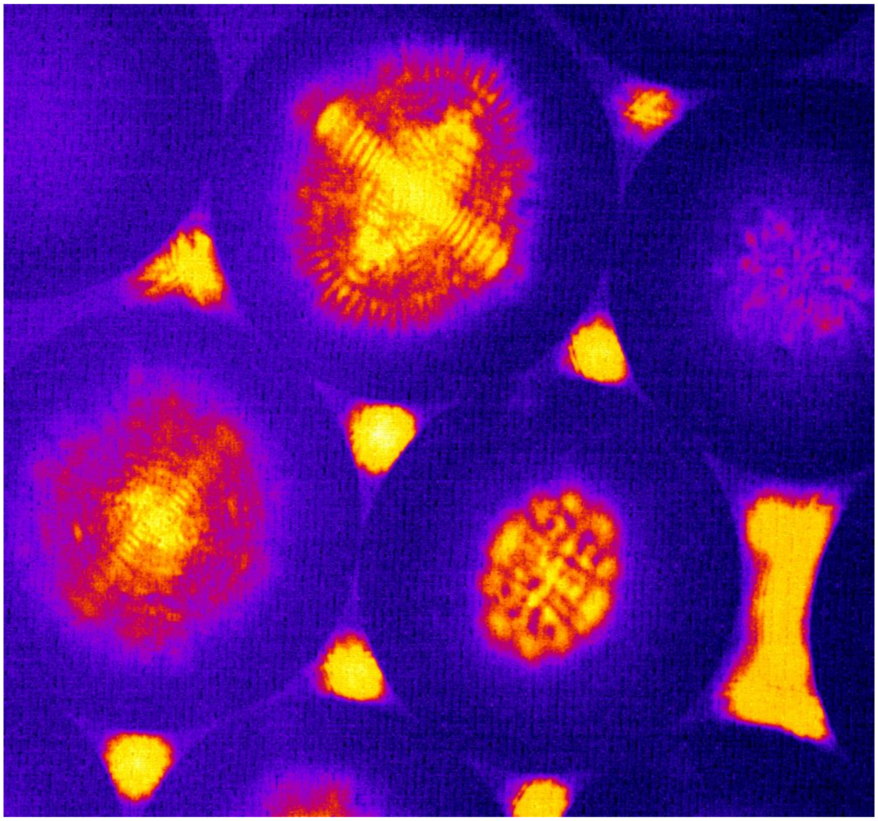

Supplementary Figure 2: IG and fractal laser modes emitted from a portion of an array of microspheres.

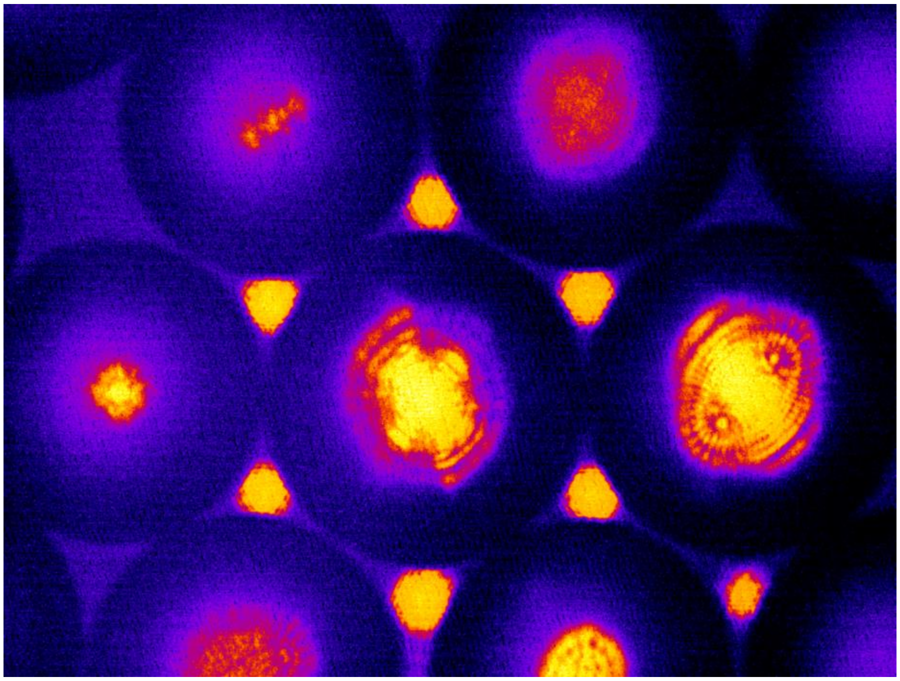

Supplementary Figure 3: Optical micrograph (false color) of both IG and fractal laser modes. The latter are generated within interstitial regions.

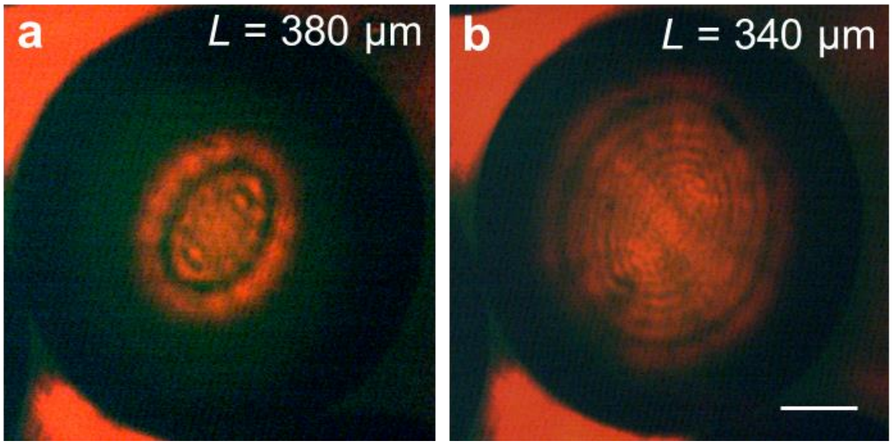

Supplementary Figure 4: Increase in IG mode order as L is decreased. a, IG transverse mode observed from a single sphere when L is set at 380 μm. **b**, Mode pattern observed from the same sphere when L is reduced to 340 μm. The scale bar width is 40 μm.

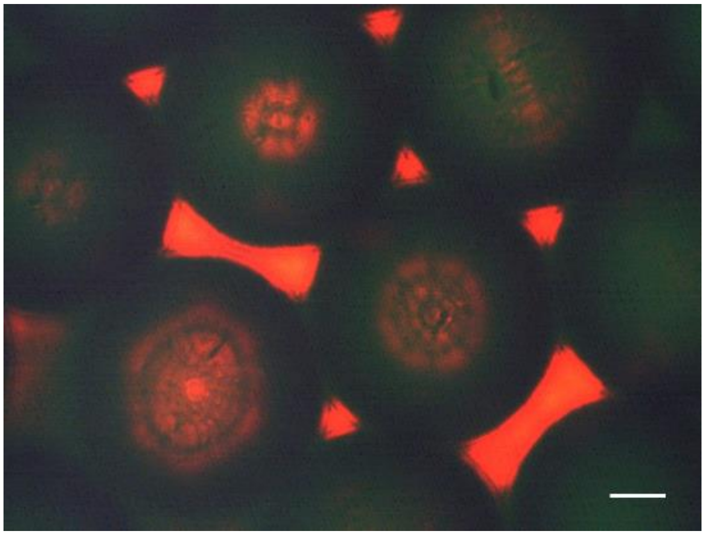

Supplementary Figure 5: Another optical micrograph of a portion of a microsphere array, showing low-order fractal modes similar to that of Fig. 3(b) in 5 interstitial regions. Coupled fractal modes and higher-order IG_{mn} modes are also visible.

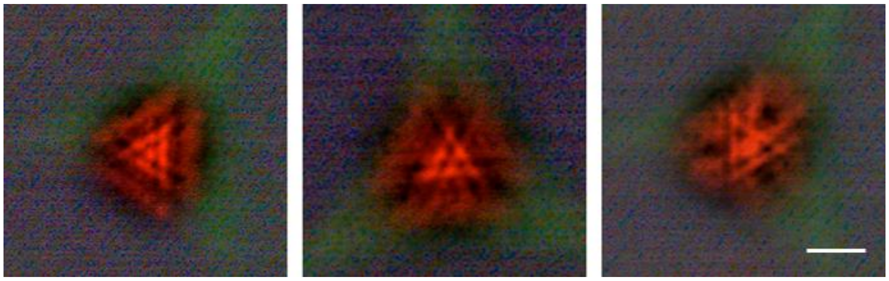

Supplementary Figure 6: An assortment of other fractal mode images (scale bar: 12 μm).

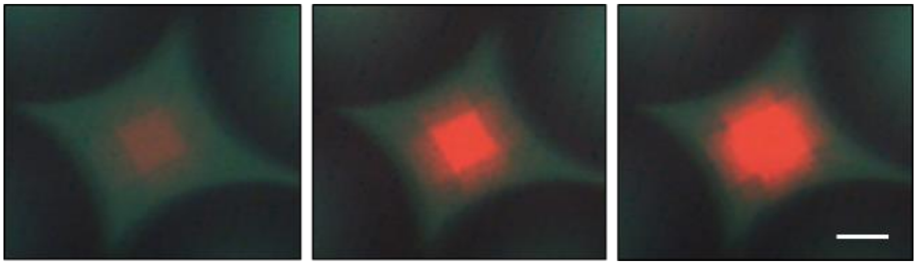

Supplementary Figure 7: Optical micrographs, acquired with a 10x objective, for interstitial regions formed by 4 microspheres (scale bar: 20 μm).

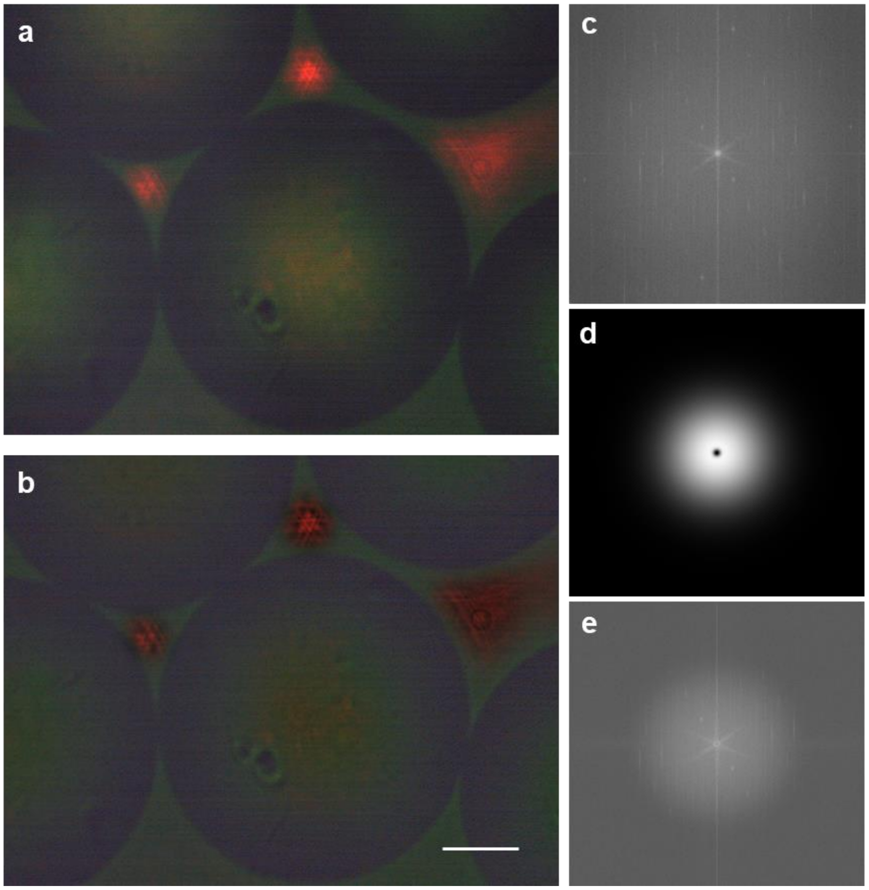

Supplementary Figure 8: Images acquired with a 20x objective are processed by implementing a bandpass filter[1] in the frequency domain using ImageJ software. a, Raw image captured by a CCD camera. **b,** Resulting image after implementing a bandpass filter in the Fourier domain. **c,** FFT of the raw image from panel **a. d,** Image of the bandpass filter. **e** Resulting FFT of the processed image.

Supplementary References

1. Gonzalez, R.C. & Woods, R.E. *Digital Image Processing* 2nd ed. (Prentice Hall, 2008).
