# Supplementary material for: Fractal modes and multi-beam generation from hybrid microlaser resonators
Source: Nat Commun. 2018 Jul 3;9:2594. doi: 10.1038/s41467-018-04945-8 (PMC6030083; doi:10.1038/s41467-018-04945-8)
Supplement: Supplementary file 3 — Description of Additional Supplementary Files [file 41467_2018_4945_MOESM3_ESM.pdf]

Description of Additional Supplementary Files

File Name: Supplementary Movie 1

Description: Supplementary Movie 1 shows a single C. reinhardtii cell being drawn to green pump radiation by its eyespot, and lasing occurs as long as the cell remains within the pump field.
