# Supplementary material for: Fractal modes and multi-beam generation from hybrid microlaser resonators
Source: Nat Commun. 2018 Jul 3;9:2594. doi: 10.1038/s41467-018-04945-8 (PMC6030083; doi:10.1038/s41467-018-04945-8)
Supplement: Supplementary file 2 — Peer Review Report [file 41467_2018_4945_MOESM2_ESM.pdf]

[Editorial Note: This manuscript has been previously reviewed at another journal that is not operating a transparent peer review scheme. This document only contains reviewer comments and rebuttal letters for versions considered at Nature Communications. Mentions of the other journal have been redacted.]

Reviewers' comments:

Reviewer #1 (Remarks to the Author):

Second review of “Fractal modes and multibeam generation from hybrid microlaser resolantors” by Rivera, Galvin and Eden.
I read the authors’ reply, and I read the revised manuscript, both with considerable interest. My bottom line recommendation is to ** accept it to Nature Comm **, but with some revisions. Let me please explain.

I agree with authors that fractal modes were (so it seems) not really reported inside a LASER cavity. But at the same time – the revised manuscript still left me frustrated, for several reasons. As the authors admit, fractals have been observed in optics, including in resonators, and were reported in many publications. It seems to be true that they were not observed in a laser, and I do not underestimate the importance of this. But I am still frustrated with the inability of the authors to pinpoint clearly the novelty of this manuscript – right in the abstract and the introduction.

Are these fractals generated because of simple linear diffraction in the resonator? Or does their generation involve lasing action, most importantly, the nonlinearity of gain saturation (that is an important ingredient in any laser) and the accompanying nonlinearity in the refractive index via Kramers-Kroning (that is what gives rise to frequency pulling)?
Unfortunately, the manuscript does not address this issue at all. It describes the experiment, but we are left with the question hanging in the air: is it mostly a linear effect, arising strictly from diffraction in this special resonator and gain, or does it involve nonlinearity (gain saturation and the accompanying change in the refractive index)? If it is the former (which I suspect it is, because the word “nonlinear” does not appear anywhere in the text), this should be made clear. A paper on that is worthy of Nature Comm, but not more. If it is the NONLINEARITY that makes these fractals happen, then the work could rise to the level of [*redacted*]. But then the authors should add quite a few more citations, to the theory work of Soljacic et al, PRE 2000, Sears et al., PRL 2000, several follow up papers, and experiments on the nonlinear evolution of optical fractals in the temporal domain (Oktem et al., Nature Photonics 2010).
This important point should be made clear, so as not to confuse the laser action (which inherently involves nonlinearity) with “just” having fractals in a resonator with gain.

As for the rest, I think the references should include Berry’s 1979 paper, Berry & Klein 1996, and Mendoza-Yero 2012 paper in Optics Letters.

Reviewer #2 (Remarks to the Author):

I have read the revised manuscript, all of the original reviewer comments and the authors’ response to them. I notice that the authors naturally make an effort to counter each reviewer

comment, but what does not feel so right to me is the impression I get that they are trying to counter each with a narrow focus, which fails to capture the broader perspective when they are considered altogether. I don't think their advance is convincingly significant from an applications point of view and that the main merit is probably reporting the first experimental observations of fractal modes. This is something that Reviewer 1 thinks (incorrectly) to have been achieved previously and it is likely that he or she would find quite interesting once realizing that a clear experimental demonstration was not made. Reviewer 2 thinks is "interesting," but is not wholly convinced about its demonstration here.

Instead of focusing on this achievement, the authors are perhaps diluting their message by trying to list other achievements which are of unconvincing impact. I am particularly not convinced by the second main claim of the revised manuscript (first being the fractal modes), namely "image as well as probe unicellular organisms internal to the cavity with <10 ns temporal resolution." As I will discuss below in more detail, first, this is not the first technique that can record images with <10 ns resolution. Second, they are not really imaging in the usual sense; they need to digitally reconstruct the image. The frame rate is limited by their laser repetition rate of merely 10 Hz (who needs to capture an image for only nanoseconds in duration, but at Hz repetition rate?), etc. Yes, there is a potential, but unless they can observe something (anything, it does not even have to be particularly interesting from a biological point of view) happening to or within the cell with such temporal resolution, I do not believe it is fair at all to claim they can image with such resolution. There is at best a potential and one of very unclear utility.

Overall, I believe the main merit of this study is not applications, because, I repeat, none have been clearly demonstrated, nor even very plausibly argued. The main appeal of this manuscript is on the fundamental side, i.e, the claim of first experimental observation of fractal modes. However, on that front, Reviewer 2 has very reasonable concerns and those concerns have been dismissed by the authors without to-the-point answers. I was especially surprised that they have selectively quoted Reviewer 2, electing to skip the most relevant remarks.

My assessment for the manuscript is that I am not convinced by any applications potential at all and nothing concrete is being reported at present. I might recommend publication on the grounds that this is the first experimental demonstration of fractal modes, and only if they respond to the concerns of Reviewer 2 and make a very compelling case that they have indeed observed fractal modes. I am not sure what fractal modes are good for, I mean even the fundamental implications are not at all. What do we learn that we did not know by this observation? Does have any implications for optics beyond this particular system? Such questions can be asked, but at least, they are "interesting" in their own right. This might enough to merit publication in Nature Communications, but I would like a very clear, convincing demonstration that we are indeed seeing self-similarity over multiple scales. Repetition (self-similarity) over multiple scales is the defining feature of a fractal: At present, the triangular shape appears to be repeated only once and even that requires some squinting and goodwill on part of the reader to recognize. One repetition is not convincing enough to claim a fractal in any case. Can they improve their experiment or its analysis to show that it continues to repeat at least one more order?

Below, I will systematically revisit some of the reviewer points and the authors' responses.

Responses to Reviewer 1's Comments:

- Regarding the absence of an experimental demonstration of fractal modes, the authors appear to be correct when they say "… the results we report in the present manuscript represent the first observation of laser fractal modes."

I believe this might be the central claim of this manuscript. All other arguments appear quite weak in my opinion. Please see below in the context of Reviewer 2's comment on the demonstration of fractal modes. Is the present demonstration strong enough? I am not so sure.

- Next, I would like to comment on the following remarks by the authors where they argue that their technique can be used for imaging at a temporal resolution that it is increased by “at least four order of magnitude”:

[*redacted*]

Now, this [*Editorial Note: dynamic imaging of living cells*] is a very shaky claim. First, imaging techniques based on using even femtosecond pulses which illuminate a camera have been around for a long while and has been used in various studies. Here, the camera is kept in the dark except only when a laser pulse (sometimes as supercontinuum in order to reduce speckle patterns) is turned on. Thus, even though the camera’s electronics are very slow, the recording is only during the pulse and the pulse duration sets the timescale. This is, then, repeated at the repetition rate of the laser, limited by the frame rate electronics the camera. For onboard memory storage, high-end cameras can record frames with microsecond rates, corresponding to kHz or even MHz frame rates, where each frame can capture with femtosecond or picosecond resolution (set the laser pulse). So, such techniques exist. I do not know if those techniques are actively used for microscopy of living organisms (they are being used for plasma studies, laser material processing, etc), but we have a nearly MHz-speed camera in my lab, that we already use to record a limited (by onboard memory storage) number of images. It is not “5 ns” resolution, and its spatial resolution is bad due to a technique called binning, but it is a commercial device. So, it is simply not correct to say they increase temporal resolution by four orders of magnitude.

Furthermore, the authors merely argue this possibility without even describing how they would do a realistic imaging scenario (frame limitations). There is absolutely no such demonstration in the manuscript. Besides, in their case, the geometry is restricted to using this array of microspheres, the spatial resolution is not clear, the repetition rate is merely 10 Hz, the images have to reconstructured. They are welcome to propose this as a potential application, but I believe a lot more restraint is warranted before claiming: “Consequently, it is now possible to image living cells on a time scale that permits the observation of dynamic behavior.” I am sorry you cannot claim this yet. You can, at best, claim possible applications in this direction. Or, actually obtain the results, revise the manuscript, and I believe it would be a great addition.

Overall, the authors appear to overplay their hand when it comes to applications, repeatedly in the response letter and the manuscript. Frankly, there is NONE here in this manuscript. There are potential ones that they argue, and even their potential is not so clear to me. If they beg to differ, they need to demonstrate them. At least a step forward.

Responses to Reviewer 2’s Comments:

[*redacted*]

Well, it should have been quite clear why Reviewer 2 made those remarks. The laser’s technical performance is dismal in comparison to modern lasers due to its extremely low efficiency and need for high-energy pulses to even operate. The authors go on to explain that these parameters are typical for similar lasers pulsed on the nanosecond scale, but that is a circular argument: The reviewer did not say that at the hand’s of these authors, this nanosecond-pulsed laser is underperforming. Rather, it was a general remark that the technical capabilities of the laser are completely underwhelming in comparison to the state of the art in lasers so if it will have applications, they can likely be niche applications. The larger issue is whether this demonstration has any clear potential for applications or not. If there is, the authors are not making a compelling case for it, not in the original and not in the revised manuscript. They list the following, e.g., as possibilities:
[*redacted*]

Well, this is very non-specific. How will it be interesting to optical imaging and in what way better than the plethora of existing sources? The beams have arbitrary phases, and I believe that is a very major shortcoming. The authors just wave off this issue, claiming that control of their phases is readily achievable. How on earth do they propose to this? If they demonstrate that, the potential impact of the paper would go up tremendously.

In short, I am not impressed by the potential of having mutually random phased beams, however many they are. Furthermore, it is a fact that they are extremely weak and inefficient.

Continuing on, the authors state “We should also add that Reviewer #2 is incorrect in saying that this laser ‘….requires pulse pumping by a 8-Hz nanosecond laser..’ This is not so- the pulsed laser was chosen to demonstrate the temporal characteristics of which this laser is capable.”

Well, if the authors can repeat these results with a much higher duty-cycle (much longer-pulsed and/or much higher repetition rate) laser, again, that would address this reviewer’s concerns to a great extent. Simply claiming that their choice of the present laser was for “demonstration” is not convincing. I believe they would have horrible problems with increased pulse energy or increased average power, particularly with living cells along the way. The scalability is not clear at all. If it is there, it should be argued clearly, rather than being hand-waved.

I get the feeling that the authors are trying to strike down each reviewer comment without paying attention to whether they can be specific, concrete about their counter-arguments and whether they can back them up. I’d like to give the benefit of doubt, maybe they are right, but the responses do not sound convincing.

- In response to the following reviewer points:
[*redacted*]

I find the selective quotation to be a little inappropriate and misleading, because the reviewer explains the issue precisely, namely that the self-similarity is not demonstrated clearly, whereas the quoted part gives the impression of unfounded complaint on the part of the reviewer. Furthermore, the authors respond by saying that they are [*redacted*] (one of their many expressions of surprise in the response letter). Well, I do not believe they should be surprised if one reads the entire remark of the reviewer. A fractal’s defining feature it is repetition over multiple scales. The authors’ response is sidestepping this central question. I remain unconvinced by their response. Furthermore, does the supposedly fractal formation imply some limited amount of coherence arising, perhaps among only adjacent microspheres? Can the authors resolve the fractal at a small scale to show it repeats itself at least once more? These are valid questions, I believe.

Reviewer 2 had several other remarks, which were not responded to.

[*redacted*]

I would appreciate if the authors respond to these as well.

Reviewer 3’s Comment

The authors respond to Reviewer 3 by listing three potential applications:
[*redacted*]

The first [*Editorial note: produce large arrays of fractal microlasers*] is not an application in itself.

The second [*Editorial note: imaging of life cells*]: How is this an interesting technique for imaging cells? It is unclear to me. There are many advanced optical microscopy techniques. Is there anything that the demonstrations of this manuscript can do better? Even potentially? If yes, it is not discussed here. Merely stating that can use this system for imaging does not constitute an application. I guess the authors would mention nanosecond resolution, but it should be demonstrated. They show us a dynamic behavior on the nanosecond timescale, if they want to claim this.

The third [*Editorial note: LIDAR and microscopy*]: Again, very non-specific. With this dismal efficiency and power level? With all the advances in beam and mode shaping, including powerful devices such as spatial light modulators, deformable mirrors, and many others? If, as remarked by Reviewer 2, the authors could demonstrate phase locking or even control of phases between their many beams, yes, there is an interesting potential, but despite the authors claim that this is "readily achievable" it is not demonstrated.

I agree with the authors that there can also be merit based on fundamental significance, as expressed at the beginning of my report. I think the authors should focus on this aspect.

RESPONSE TO REVIEWERS: MANUSCRIPT NO. NCOMMS-17-31592-T

REVIEWER #1

1. "My bottom line recommendation is to 'accept it to Nature Comm.', but with some revisions."

We are grateful to Reviewer #1 for this statement.

2. "I agree with authors that fractal modes were (so it seems) not really reported inside a LASER cavity. But at the same time – the revised manuscript still left me frustrated for several reasons……. It seems to be true that they were not observed in a laser, and I do not underestimate the importance of this. But I am still frustrated with the inability of the authors to pinpoint clearly the novelty of this manuscript…."

   "Are these fractals generated because of simple linear diffraction in the resonator? Or does their generation involve lasing action, most importantly, the nonlinearity of gain saturation….?"

We very much regret having frustrated Reviewer #1 but we do understand why this has occurred, and I accept the responsibility for not having discussed this subject more fully in previous versions of the manuscript. As a result of his/her comments, we have carried out more extensive Fresnel diffraction calculations for our resonator and, as stated in the cover letter to Dr. Meinzer, we find that the simulations reproduce well at least one of the observed fractal modes. In fact, two of the modes observed repeatedly in the most recent round of experiments closely resembles the Sierpinski Sieve (Triangle). Because of the ability of diffraction calculations to reproduce one of the observed modes, the mechanism(s) primarily responsible for fractal mode generation is presumed to be linear. However, we are unable to dismiss the possible contribution of nonlinear effects and this will be further discussed below. Also, we do not expect gain saturation to play a significant role in these experiments. Calculations indicate that the stimulated emission cross-section for this gain medium is approximately 2×10^{-16} cm^2. Because the quantum dot solution produces a homogeneously-broadened gain profile, we calculate the saturation intensity to be roughly 36 kW/cm^2. Since the intracavity intensities are well below this value, the laser is operating in the small-signal regime and gain saturation is slight.

Having said the above, however, we note that we have been able to identify 4 basic fractal modes and the laser is robust in the sense that, for any given interstice in the microsphere network, the

fractal mode generated within is reproducible on a shot-to-shot basis. However, since the pump laser is pulsed and the peak intensity varies by a few percent with each pulse, the index of refraction varies both spatially and temporally because of the optical Kerr effect. Consequently, we are not able to be dogmatic in suggesting that the fractal generation process is linear, and further experiments and calculations are necessary to resolve this issue.

Another aspect of the novelty of the manuscript (mentioned above by Reviewer #1) is that the intracavity "apertures" in the resonator are interstitial regions in the microsphere array that are immersed in the gain medium. Consequently, interference fringes produced by microsphere diffraction of the pump result in spherical surfaces of maximum gain within the interstices. These surfaces and their intersections form a scaffold from which the fractal modes are constructed. This is seen clearly in the images of Figs. 2 and 3 in the revised manuscript. We have recently confirmed that this scenario is correct by replacing the microsphere network with an array of hemispherical microlenses. In otherwise identical experiments, the fractal modes are not produced. This strongly suggests that Fresnel diffraction, and the spherical gain surfaces produced, are partially responsible for the formation of fractal modes.

The revised manuscript addresses all of the issues discussed above and we hope that Reviewer #1 finds the treatment to be acceptable. Additional references on the subject of optical fractals have also been added.

REVIEWER #2

Because the comments of Reviewer #2 are lengthy, we will not quote his/her text in full, but rather excerpts that appear to be representative:

1. "I don't think their advance is convincingly significant from an applications point of view and that the main merit is probably reporting the first experimental observation of fractal modes."

   "Instead of focusing on this achievement, the authors are perhaps diluting their message by trying to list other achievements which are of unconvincing impact."

We are in agreement with Reviewer #2. Although this was not our intention, we realize that his/her perception is reasonable. Therefore, although we continue to be convinced that the applications of this resonator are quite significant, we have trimmed back on the discussion of imaging and its potential. This has allowed us to devote more text to the observation of laser fractal modes. We are indebted to Reviewer #2 for these comments.

2. "I might recommend publication on the grounds that this is the first experimental demonstration of fractal modes, and only if they respond to the concerns of Reviewer 2 and make a very compelling case that they have indeed observed fractal modes. I am not sure what fractal modes are good for. I mean even the fundamental implications are not at all [sic]….. I would like a very clear, convincing demonstration that we are indeed seeing self-similarity over multiple scales."

We also appreciate these comments by Reviewer #2, and have addressed the issues raised here in our comments to Reviewer #1 and the cover letter to the Editor. Specifically, a large set of images recorded at 20x magnification show self-similarity clearly, and should lay to rest any concerns regarding the fractal nature of the observed laser modes. One of the observed modes, for example, is very similar to the Sierpinski Sieve (or Triangle) which is a well-known, exact fractal. Fresnel diffraction calculations also match one observed fractal mode well.

3. “Well, it should have been quite clear why Reviewer #2 made those remarks. The laser’s technical performance is dismal in comparison to modern lasers due to its extremely low efficiency and need for high-energy pulses to even operate.”

Although we appreciate the majority of the comments of Reviewer #2, this statement compels us to respond. In particular, this statement by Reviewer #2 betrays a lack of familiarity with laser development. The reviewer is apparently unaware that every single “modern laser” performed dreadfully when first operated. Please forgive the personal example but my most extensive experience is with the excimer lasers (first demonstrated in 1975 and 1976) that are now being used worldwide for photolithography, and medical procedures and therapy (LASIK, etc.). I can say emphatically that their efficiencies were initially “dismal”, lower than that for the laser reported here, beam quality was beyond poor, and they produced only a few pulses before lasing ceased entirely. Only after years of development did the potential of these lasers become evident and commercial products were feasible. An almost identical account can be given for all “modern lasers”: carbon dioxide, Ti:sapphire, fiber, Nd:YAG, and semiconductor lasers (edge-emitters and VCSELs). I am familiar with the details of the development of each of these and can say that the performance of every single one of them was initially “dismal”.

I recognize that making a prediction regarding the ultimate utility of any laser is risky, but our experience suggests that retaining the structure of the resonator reported here but changing the gain medium (quantum dots were adopted initially for convenience) and the use of an integrated master oscillator/power amplifier (MOPA) will make feasible a multibeam laser with overall pump-to-output conversion efficiencies of tens of percent. Continuous wave (CW) operation will also certainly be achieved once the structure of this resonator is broadly known.

4. “In short, I am not impressed by the potential of having mutually random phased beams, however many they are. Furthermore, it is a fact that they are extremely weak and inefficient.”

I was remiss for not having elaborated on the potential value of an array of microlasers “having mutually random phased beams.” Although these results are preliminary at present, we can say that these arrays provide a high quality, speckle-free laser source, precisely because the phases of each of the beams varies randomly with respect to those of the others. In hindsight, we should have expected this result because it is intuitively true. Because Reviewer #2 refers earlier to the ability of supercontinua to “reduce speckle patterns”, perhaps he/she is familiar with the effort devoted over the past two decades to developing speckle-free lasers. Mechanical solutions and “random” lasers are two avenues that have been pursued, but neither is satisfactory in the long run. Since we are not yet prepared to offer a significant amount of data in this area, we have inserted a modest amount of text into the revised manuscript.

The points discussed above appear to be the primary objections and concerns raised by Reviewer #2. It is our hope that each has been addressed adequately. In each case, the discussion of the topic in the revised manuscript has been modified, and we hope that Reviewer #2 finds the revisions to be acceptable.

REVIEWERS' COMMENTS:

Reviewer #1 (Remarks to the Author):

I recommend to accept the paper.
I read the report of the other referee - and I strongly disagree with many of his/her statements. The applications of a newly found physical phenomenon, whether such applications exist or not, is far less important when the discovery is made. Times and again the applications of a new discovery are suggested only years later, and have a real impact in industry only after decades.

I believe that a new phenomenon deserves publication on it's own merit, irrespective of whether or not it has applications.

This manuscript should be accepted, period.

Reviewer #2 (Remarks to the Author):

The authors have responded positively to the suggestion to emphasize the first experimental observation of fractal modes and have further significantly advanced the experimental evidence for the existence of these modes. I believe the manuscripts is now more focused and makes well-substantiated claims.

I support its publication in this form.

I do have one comment, which I would like to pass on as "optional" in order not to delay the publication decision:

There are several very vague statements regarding the possible role of nonlinear effects. The authors write (page 4):
"Although the primary mechanism responsible for generating the fractal modes appears to be linear in the pump optical field intensity, the influence of nonlinearities cannot be discounted."
There is a similarly vague discussion on page 12:
"However, it is not possible at present to dismiss the influence of a nonlinear process, ..."

The authors only mention "gain saturation" as a possible nonlinear mechanism, but they also write: "its impact is small." So, nothing remains at hand.

While it is indeed possible that nonlinear effects play a role, in the absence of anything more concrete, these vague remarks do not contribute positively to the manuscript. I would like to suggest that the authors add a single, shorter comment more appropriate to the situation if they feel strongly about it or remove these statements entirely.

RESPONSE TO REVIEWERS: MANUSCRIPT NO. NCOMMS-17-31592-T

REVIEWER #1

> "I recommend to accept the paper. I read the report of the other referee – and I strongly disagree with many of his/her statements. The applications of a newly found physical phenomenon, whether such applications exist or not, is far less important when the discovery is made. Time and again, the applications of a new discovery are suggested only years later.
>
> I believe that a new phenomenon deserves publication on its own merit, irrespective of whether or not it has applications.
>
> This manuscript should be accepted, period."

We are overwhelmed by the comments of Reviewer #1 and do not know how to respond other than to say: "THANK YOU!"

REVIEWER #2

1. "The authors have responded positively to the suggestion to emphasize the first experimental observation of fractal modes and have further significantly advanced the experimental evidence for the existence of these modes. I believe the manuscript is now more focused and makes well-substantiated claims.

   I support its publication in this form."

We are grateful to Reviewer #2 for these comments. We would also like to say that this manuscript would not have been strengthened to the extent that it has if both reviewers had not made their insightful and probing comments. We wish to express our thanks to you both!

2. "I do have one comment, which I would like to pass on as "optional" in order not to delay the publication decision:

   There are several very vague statements regarding the possible role of nonlinear effects. The authors write (page 4): 'Although …………………………'
   There is a similarly vague discussion on page 12:

> ‘However, it is not possible at present……..’
> I would like to suggest that the authors add a single, shorter comment more appropriate to the situation if they feel strongly about it or remove these statements entirely.”

We appreciate this suggestion of Reviewer #2. The origin of this addition to the manuscript was several comments made by Reviewer #1 concerning the potential influence of nonlinear optical mechanisms on the generation of fractal laser modes. Because we believe Reviewer #1 to be correct, we made several calculations regarding gain saturation and, after some thought, decided to add the text quoted by Reviewer #2.

It is our conviction that we cannot ignore the possible impact of nonlinear processes on our experiments. Although the absence of extensive experimental data in this area at present prevents us from commenting extensively, we believe we have an obligation to mention this topic, if only briefly. Consequently, we have decided to retain most of the text on this subject that was introduced at the last revision, but we have slightly modified the language. It is our hope that Reviewer #2 finds this to be acceptable.
